# Supplementary material for: Exploring stakeholder perceptions of peer support initiatives in the management of diabetes in low- and middle-income countries: An online survey study
Source: PLOS Glob Public Health. 2026 Feb 5;6(2):e0005840. doi: 10.1371/journal.pgph.0005840 (PMC12875572; doi:10.1371/journal.pgph.0005840)
Supplement: S1 Appendix — (DOCX) [file pgph.0005840.s001.docx]

**S1 Appendix**. Income Level and Number of Respondents by Country

| Country | Income Level | Number |
| --- | --- | --- |
| Afghanistan | Low | 2 |
| Palau | Low | 1 |
| Malawi | Low | 4 |
| Ethiopia | Low | 1 |
| Burkina Faso | Low | 1 |
| Liberia | Low | 2 |
| Nepal | Lower Middle | 4 |
| Tunisia | Lower Middle | 2 |
| Sudan | Lower Middle | 3 |
| Rwanda | Lower Middle | 2 |
| Pakistan | Lower Middle | 3 |
| Nigeria | Lower Middle | 2 |
| Angola | Lower Middle | 1 |
| Zimbabwe | Lower Middle | 2 |
| Kenya | Lower Middle | 9 |
| Iraq | Lower Middle | 3 |
| India | Lower Middle | 2 |
| Tanzania | Lower Middle | 3 |
| Malaysia | Upper Middle | 1 |
| Brazil | Upper Middle | 7 |
| Albania | Upper Middle | 4 |
| Guyana | Upper Middle | 1 |
| Argentina | Upper Middle | 1 |
| Uganda | Upper Middle | 4 |
| Botswana | Upper Middle | 4 |
| Total |  | 69 |
