# Supplementary material for: Exploring stakeholder perceptions of peer support initiatives in the management of diabetes in low- and middle-income countries: An online survey study
Source: PLOS Glob Public Health. 2026 Feb 5;6(2):e0005840. doi: 10.1371/journal.pgph.0005840 (PMC12875572; doi:10.1371/journal.pgph.0005840)
Supplement: S2 Appendix — (DOCX) [file pgph.0005840.s002.docx]

**S2 Appendix.** Respondents with 'other' as job title

| Respondent# - other job title response | Other - text |
| --- | --- |
| ## | Rector |
| ## | Teacher |
| ## | Medical Director of International Health and Social Justice (NGO) |
| ## | Clinical Officer |
| ## | Clinical Officer |
| ## | Health Advisor |
| ## | Chief Executive Officer |
| ## | Non Communicable Disease Clinical Coordinator |
| ## | Medical Director of Health and Social Justice (NGO) |
| ## | Monitoring & Evaluation Manager |
| ## | Diabetes Peer-Educator |
| ## | Program Officer (NGO) |
| ## | Lecturer in Pharmacy |
| ## | Public Health worker |
