# Supplementary material for: Exploring stakeholder perceptions of peer support initiatives in the management of diabetes in low- and middle-income countries: An online survey study
Source: PLOS Glob Public Health. 2026 Feb 5;6(2):e0005840. doi: 10.1371/journal.pgph.0005840 (PMC12875572; doi:10.1371/journal.pgph.0005840)
Supplement: S3 Appendix — (DOCX) [file pgph.0005840.s003.docx]

**S3 Appendix.** Respondents with 'other' as type of health care facility

| Respondent# - other job title response | Other - text |
| --- | --- |
| ## | . |
| ## | County level Non Communicable Diseases Office |
| ## | Public Health Institution |
| ## | Integrated Healthcare Institution (Secondary, Primary and Faith-Based) |
| ## | Physiology Education Office |
| ## | Integrated Primary and Secondary Public Health Service Institution |
