# Supplementary material for: Exploring stakeholder perceptions of peer support initiatives in the management of diabetes in low- and middle-income countries: An online survey study
Source: PLOS Glob Public Health. 2026 Feb 5;6(2):e0005840. doi: 10.1371/journal.pgph.0005840 (PMC12875572; doi:10.1371/journal.pgph.0005840)
Supplement: S4 Appendix — (DOCX) [file pgph.0005840.s004.docx]

**S4 Appendix.** Readiness perception of key stakeholders toward T2DM peer support initiatives

|  | Mean | Std. err | [95% conf. interval] | |
| --- | --- | --- | --- | --- |
| Q-“Needs identified” | 2.320755 | .1782303 | 1.96311 | 2.6784 |
| Q-“Staff training” | 2.415094 | .1913097 | 2.031203 | 2.798985 |
| Q-“Leadership willingness” | 3.45283 | .1583799 | 3.135018 | 3.770643 |
| Q-“Trained staff” | 2.415094 | .1987515 | 2.01627 | 2.813918 |
| Q-“Funding plan” | 2.226415 | .160825 | 1.903696 | 2.549134 |
| Q-“Time and materials” | 2.735849 | .1869295 | 2.360748 | 3.11095 |
| Q-“Sustainability plan” | 2.679245 | .1975422 | 2.282848 | 3.075643 |
| Q-“Evaluation Plan” | 2.113208 | .1608675 | 1.790403 | 2.436012 |
