# Supplementary material for: Exploring stakeholder perceptions of peer support initiatives in the management of diabetes in low- and middle-income countries: An online survey study
Source: PLOS Glob Public Health. 2026 Feb 5;6(2):e0005840. doi: 10.1371/journal.pgph.0005840 (PMC12875572; doi:10.1371/journal.pgph.0005840)
Supplement: S5 Appendix — (DOCX) [file pgph.0005840.s005.docx]

| **S5 Appendix. Example of coding process for thematic content analysis** | | | |
| --- | --- | --- | --- |
| **Items** | **Raw response** | **Initial codes** | **Final theme** |
| Definition of peer support | - Patients helping each other through shared experiences. - Individuals with diabetes offering mutual support and understanding. - Support that comes from people facing similar challenges. | - Emotional support; practical help | - Mutual support and understanding |
| Definition of peer support | - Using personal experience to guide and educate others with the same condition. - Peers exchanging practical advice based on lived experience. - Learning from others who have successfully managed their condition. | - Experience sharing; coping strategies | - Experiential knowledge sharing |
| Definition of peer support | - Organized efforts to raise awareness and advocate for people with diabetes. - Community groups working together to promote access to care. - Supporting patients’ rights and building stronger health network. | - Social support network; advocacy | - Community advocacy and rights |
| Barrier to implementation | - We do not have enough funds or proper equipment. - We lack trained staff and materials. | - Lack of funding; resource constraints | - Limited resources and funding |
| Barrier to implementation | - There are no clear guidelines on how the program should work. - We are unsure who qualifies or what kind of support to provide. | - Lack of structure; unclear program objectives | - Poor program functioning |
| Factilitator of implementation | - We receive ongoing funding from UNICEF. - The World Diabetes Foundation helps sustain our activities. - External grants have kept our program alive. | - Financial assistance; sustainability | - Financial support from organizations |
| Factilitator of implementation | - Local leaders help us spread information about diabetes. - Religious groups assist in sharing management tips. | - Community mobilization; local engagement | - Community resilience |

|  |
| --- |

**Note:** Table provides representative examples illustrating how raw survey responses were coded and abstracted into broader themes.
