# Supplementary material for: Exploring stakeholder perceptions of peer support initiatives in the management of diabetes in low- and middle-income countries: An online survey study
Source: PLOS Glob Public Health. 2026 Feb 5;6(2):e0005840. doi: 10.1371/journal.pgph.0005840 (PMC12875572; doi:10.1371/journal.pgph.0005840)
Supplement: Attachment — Submitted filename: Response to Editor.docx [file pgph.0005840.s007.docx]

Dear Ms. Dimayuga and the PLOS Global Public Health Editorial Team,

**Subject:** Resubmission of Manuscript PGPH-D-24-02647

Thank you for your continued review and for providing clear guidance on the remaining items needed for our manuscript titled ‘Exploring Stakeholder Perceptions of Peer Support Initiatives in the Management of Diabetes in Low- and Middle-Income Countries: An Online Survey Study.’ We have carefully addressed the points raised and provide the following responses:

1. **Human participants research checklist:** We have completed and uploaded the Human Participants Research Checklist, as requested. We would like to clarify that our study does not meet the criteria for human subjects research, as confirmed by the Mass General Brigham Institutional Review Board (IRB). A formal letter from the IRB indicating this determination has been included with our resubmission.

Additionally, the following statement has been added under the Ethics and Consent section on page 5 of the updated manuscript:

*‘This study involved anonymous, voluntary participation in an online survey with no collection of identifiable information. Based on these characteristics, the Mass General Brigham Institutional Review Board determined that the project does not constitute human subjects research and waived the requirement for formal IRB approval (REDCap ID#: 3211)’*

2. **Resubmission of PGPH-D-24-02647:** We confirm that this manuscript is a resubmission of PGPH-D-24-02647. The manuscript has been updated to incorporate the required ethics information and checklist documentation in alignment with your submission standards.

We are grateful for your support in guiding this manuscript through the editorial process and remain available to address any further clarifications or revisions required. Thank you for your time and consideration.

With best regards,
**Dr. Bishal Gyawali**
On behalf of all co-authors
