# Supplementary material for: Exploring stakeholder perceptions of peer support initiatives in the management of diabetes in low- and middle-income countries: An online survey study
Source: PLOS Glob Public Health. 2026 Feb 5;6(2):e0005840. doi: 10.1371/journal.pgph.0005840 (PMC12875572; doi:10.1371/journal.pgph.0005840)
Supplement: Attachment — Submitted filename: Point-by-point -responses.docx [file pgph.0005840.s008.docx]

**Editor’s comments**

| **Comments** | **Responses** |
| --- | --- |
| 1. Please ensure that your Ethics Statement is available in its entirety at the beginning of your Methods section, under a subheading 'Ethics Statement'. | Thank you for your suggestion. We have updated the Methods section to include the full Ethics Statement under the subheading “Ethics Statement” on page 3. |
| 2. We note that your Data Availability Statement is currently as follows: “All relevant and available data is included in this paper.”  Please confirm at this time whether or not your submission contains all raw data required to replicate the results of your study. Authors must share the “minimal data set” for their submission. PLOS defines the minimal data set to consist of the data required to replicate all study findings reported in the article, as well as related metadata and methods ([https://journals.plos.org/plosone/s/data-availability#loc-minimal-data-set-definition](https://journals.plos.org/plosone/s/data-availability#loc-minimal-data-set-definition)). | We have added .xls data file containing all relevant raw data required to replicate the results of the study (see the supplementary file) |
| 3. Some material included in your submission may be copyrighted. According to PLOS’s copyright policy, authors who use figures or other material (e.g., graphics, clipart, maps) from another author or copyright holder must demonstrate or obtain permission to publish this material under the Creative Commons Attribution 4.0 International (CC BY 4.0) License used by PLOS journals. Please closely review the details of PLOS’s copyright requirements here: PLOS Licenses and Copyright. If you need to request permissions from a copyright holder, you may use PLOS's Copyright Content Permission form. | All image and figures in this manuscript are original, including Figure 1. There are no copyrighted materials or third-party graphics used. All plots were generated using STATA/BE version 17.0 and Python 3.12, using the packages geopandas, pandas and matplotlib. |
| 4. Please provide a direct link to the base layer of the map (i.e., the country or region border shape) and ensure this is included in the figure legend; and provide a link to the terms of use / license information for the base layer image or shapefile. | The map was generated using the open-source Python package **GeoPandas** (version 0.2.1; <https://geopandas.org/>). The underlying shapefile used by GeoPandas’ built-in datasets is derived from Natural Earth (public domain; <https://www.naturalearthdata.com/about/terms-of-use/>). No proprietary or copyrighted sources (e.g., Google Maps, MapQuest) were used. |

**Reviewer’s comments**

| **Comments** | **Responses** |
| --- | --- |
| **A. Strength of the study are,** • Peer support for T2DM management in LMICs is a relatively underexplored area, • Application of the SELFIE framework provides a strong theoretical and conceptual base. • Survey design, domains, sampling strategies, and administration are clearly reported, with transparency ensured through the CHERRIES checklist. • Data collection spanning 25 LMICs enhances the breadth and generalizability of the study. • Inclusion of varied healthcare settings—tertiary, secondary, primary, and private facilities—adds contextual depth and strengthens the findings. | Thank you for your positive feedback. We appreciate your recognition of our study and are encouraged by your comments. |
| **1. Sample Size Justification:** The manuscript does not describe any attempt to calculate or justify the required sample size. Without a priori sample size estimation, it is unclear whether the study was adequately powered to capture the diversity of stakeholder perceptions across LMICs. | We acknowledge the reviewer's important comment regarding sample size justification. A traditional *a priori* calculation was not conducted as this was an exploratory, descriptive study without a primary hypothesis to test; its key aim was to map the landscape of perceptions across a diverse range of LMICs, for which parameters for a power calculation were unavailable. Instead, our sampling strategy was guided by the principle of achieving breadth and diversity of perspectives. Our final cohort of 69 respondents from 25 different LMICs, representing macro, meso, and micro levels of the healthcare system, successfully captured a wide variety of insights and allowed for the identification of consistent, emergent themes, particularly in the qualitative data, indicating that the sample was sufficient for the study's exploratory objectives. We have now explicitly addressed this justification in the manuscript's methods section (please refer to page 5 of the manuscript). |
| **2. Internal Validity:** Measures to ensure internal validity are insufficiently reported. Details regarding pilot testing of the tool and steps taken to reduce measurement error are lacking. These omissions reduce the methodological rigor. | We thank the reviewer for highlighting the need to detail measures for internal validity. Survey results are inherently subject to confounding but in our specific context, we did everything possible to maximize internal validity of the survey tool. It was initially reviewed by our multidisciplinary study team, comprising professionals from medicine, public health, pharmacy, nursing and physiotherapy, all with experience conducting research in LMICs. The instrument was then refined based on feedback from an external panel of three experts to enhance content validity and question clarity. Finally, the survey was pilot-tested with a small group of healthcare professionals and program managers who were not part of the final sample; this process confirmed the functionality of the branching logic, estimated completion time, and further improved the instrument's clarity and precision (see the ‘survey administration’ section on page 5 of the manuscript). We used the SELFIE framework and CHERRIES checklist, and no additional standardized tools exist for this area of research. |
| **3. Response Rate and Representativeness:** The authors state that only 69 respondents completed the survey. This is a very low number considering the broad geographical scope across LMICs. Such a poor response rate introduces non-response bias and raises concerns about the representativeness and generalizability of the findings. | We thank the reviewer for raising this important concern. We fully acknowledge that the sample size limits the representativeness of the findings and introduces the potential for non-response bias. As an exploratory study, its primary aim was not to achieve generalizability but to gather initial, rich insights from a strategically diverse range of stakeholders across 25 LMICs. The value of our sample lies in this qualitative diversity rather than its quantitative size. We have explicitly addressed this limitation in the manuscript, clarifying that the findings provide preliminary evidence to inform the design of larger, more representative future studies (see last paragraph on page 12 of the manuscript). We still believe that these preliminary, hypothesis generating perspectives are worth sharing and recommend that future research with additional funding should prioritize this topic using our survey tool or a similar one. In light of this reviewer comment, we have further tempered our discussion section with notes on the limitations of the study. |
| **4. Thematic Content Analysis:** Although the authors mention that thematic content analysis was used, the process of theme development is not clearly described. It is not specified whether themes emerged inductively from the data or were based on a pre-existing framework. Furthermore, no details are provided on coding procedures, inter-coder reliability, or whether data saturation was achieved. This lack of transparency undermines the credibility of the qualitative findings. | We thank the reviewer for raising this important point. To enhance methodological transparency, we have expanded the description of our qualitative analysis. The process began with an inductive review of the brief, open-ended survey responses to identify emergent concepts. These initial concepts were then systematically organized and interpreted using the integrated SELFIE framework, which provided a deductive structure for categorizing findings across macro, meso, and micro levels. This combined approach was well-suited to the concise nature of the survey data. Two researchers (BG and SB) independently coded all responses, then met to discuss and resolve discrepancies through consensus-building discussions. Although the brevity of the responses limited deeper exploration, thematic saturation was achieved for the core barriers and facilitators relevant to our study objectives. Full details of this process have been added to the methods section. Examples of how raw responses were coded and abstracted into themes are provided in *Supporting Information S5* (example of coding process for thematic content analysis). (see ‘data analysis’ section on page 6 of the manuscript). |
| **5. Sampling Bias:** A majority of the respondents were physicians, which introduces sampling bias. Other important stakeholders—such as nurses, allied health workers, policymakers, and patients—were either underrepresented or excluded. This imbalance should be explicitly acknowledged in the limitations section. | We thank the reviewer for this critical observation. We fully acknowledge this limitation. The over-representation of physicians in our sample, despite our efforts to reach a diverse stakeholder group, introduces a significant sampling bias. This likely skews the findings towards the perspectives, priorities, and clinical experiences of medical doctors, potentially underrepresenting the views of other crucial stakeholders such as nurses, allied health professionals, policymakers, and patients. This imbalance limits the generalizability of our findings across the entire healthcare ecosystem and is now explicitly discussed in the limitations section of the manuscript. (see the first para on page 13 of the manuscript). |
| **6. Depth of Qualitative Data:** The qualitative analysis appears to have been derived from brief survey responses rather than in-depth interviews. As a result, the richness and nuance of stakeholder perspectives are limited. This constitutes a methodological limitation that should be clearly highlighted. | We agree with the reviewer's assessment. The qualitative data in our study were indeed derived from brief open-ended questions within a survey, not from in-depth interviews. Unfortunately, our resources did not allow us to undertake interviews, but this inherently limits the depth and nuance of the responses, as participants did not have the opportunity to elaborate on their perspectives in detail. This constitutes a methodological limitation of our study, and we added it to the limitations section. Despite this constraint, these responses provided valuable, succinct insights into the most immediate and salient barriers (e.g., lack of funding) and facilitators (e.g., local leadership) perceived by stakeholders, which aligns with the exploratory aim of our survey. (see the first para on page 13 of the manuscript). |
| **7. Exclusion of Patients as Stakeholders:** The study excludes patients, who are central to peer support initiatives. Without including their perspectives, it is difficult to conclude that “stakeholders in LMICs” as a whole demonstrate readiness for peer support initiatives. This limits the scope and applicability of the findings, and the conclusions should be tempered accordingly | This is an important point, and we have also added this to the limitations section. Due to method of distribution of the survey (online in LMICs) reaching patients was difficult and resource limitations did not allow for special provisions to reach patients. Patient perspectives (as well as perspectives of the “peers” in peer support) is certainly an important piece of ongoing research. We have adjusted the discussion and conclusion sections to this effect. |
